# Supplementary material for: Visualizing designer quantum states in stable macrocycle quantum corrals
Source: Nat Commun. 2021 Oct 8;12:5895. doi: 10.1038/s41467-021-26198-8 (PMC8501084; doi:10.1038/s41467-021-26198-8)
Supplement: Supplementary file 1 — Supplementary Information [file 41467_2021_26198_MOESM1_ESM.pdf]

Supplementary Information
Visualizing designer quantum states in stable macrocycle quantum corrals

Xinnan Peng,[1] Harshitra Mahalingam,[2] Shaoqiang Dong,[1] Pingo Mutombo,[3] Jie Su,[1] Mykola Telychko,[1] Shaotang Song,[1] Pin Lyu,[1] Pei Wen Ng,[1] Jishan Wu,[1] Pavel Jelínek,[3,4] Chunyan Chi,[1] Aleksandr Rodin,[2,5] and Jiong Lu[1,5]

(jelinekp@fzu.cz, chunyan@nus.edu.sg, aleksandr.rodin@yale-nus.edu.sg, chmluj@nus.edu.sg)

[1]*Department of Chemistry, National University of Singapore, Singapore 117543, Singapore*

[2]*Yale-NUS College, 16 College Avenue West, Singapore 138527, Singapore*

[3]*Institute of Physics, Czech Academy of Sciences, Prague 16200, Czech Republic*

[4]*Regional Centre of Advanced Technologies and Materials, Palacký University, Olomouc 78371, Czech Republic.*

[5]*Centre for Advanced 2D Materials (CA2DM), National University of Singapore, Singapore 117543, Singapore*

Supplementary Note 1. Analytical Theory.

To describe the Au(111) surface states, we employ the single-band nearly-free electron model with the dispersion $\varepsilon_{\mathbf{q}} = \hbar^2|\mathbf{q}|^2/(2m_e m^*)$, $\mathbf{q}$ is the crystal momentum, m_e is the electron mass and $m^* = 0.27$ is the effective mass. The polymer induces a local potential so that the second-quantised Hamiltonian can be written as

$$\hat{\mathcal{H}} = \sum_{\mathbf{q}} c^\dagger_{\mathbf{q}}(\varepsilon_{\mathbf{q}} - \mu)c_{\mathbf{q}} + \sum_{\mathbf{r}} c^\dagger_{\mathbf{r}} U_{\mathbf{r}} c_{\mathbf{r}} = c^\dagger_{\mathbf{Q}} \left(H_{\mathbf{Q}} - \mu\right) c_{\mathbf{Q}} + c^\dagger_{\mathbf{R}} \mathbf{U} c_{\mathbf{R}} \,. \tag{1}$$

In the first term, $c^\dagger_{\mathbf{q}}(c_{\mathbf{q}})$ are fermionic creation (annihilation) operators associated with the single-band eigenstates and μ is the chemical potential. Because we are considering a single band, each unit cell at coordinate $\mathbf{r}$ contains a single state whose creation (annihilation) operator is $c^\dagger_{\mathbf{r}}$ $(c_{\mathbf{r}})$. $U_{\mathbf{r}}$ is the polymer-induced potential variation at $\mathbf{r}$. To make the expression more compact, we collect all $c_{\mathbf{q}}$ and $c_{\mathbf{r}}$ into vectors of operators $c_{\mathbf{Q}}$ and $c_{\mathbf{R}}$, respectively. From this, $H_{\mathbf{Q}}$ is a diagonal matrix of $\varepsilon_{\mathbf{q}}$, while $\mathbf{U}$ is a diagonal matrix of $U_{\mathbf{r}}$.

Because $c_{\mathbf{R}}$ and $c_{\mathbf{Q}}$ are related by the unitary transformation $c_{\mathbf{R}} = \Theta c_{\mathbf{Q}}$, where $\Theta_{jk} = e^{i\mathbf{r}_j\cdot\mathbf{q}_k}/\sqrt{N}$ and N is the number of states in the system. This results in

$$\hat{\mathcal{H}} = c^\dagger_{\mathbf{R}} \left[\Theta \left(H_{\mathbf{Q}} - \mu\right) \Theta^\dagger + \mathbf{U}\right] c_{\mathbf{R}} \,. \tag{2}$$

This Hamiltonian can be transcribed into the imaginary-time action

$$S = \sum_{\omega_n} \bar{\phi}_n \left[\Theta \left(H_{\mathbf{Q}} - \mu - i\omega_n\right) \Theta^\dagger + \mathbf{U}\right] \phi_n \,, \tag{3}$$

where ϕ_n is a vector of Grassmann numbers corresponding to the fermionic operators and ω_n are the fermionic Matsubara frequencies. Exponentiating the action and integrating over the fields yields the partition function

$$\mathcal{Z} = \prod_{\omega_n} \left|\beta \left[\Theta \left(H_{\mathbf{Q}} - \mu - i\omega_n\right) \Theta^\dagger + \mathbf{U}\right]\right| = \prod_{\omega_n} \left|\beta \left(-\Xi^{-1}_{i\omega_n+\mu} + \mathbf{U}\right)\right| = \prod_{\omega_n} \left|-\beta G^{-1}_{i\omega_n+\mu}\right| \,, \tag{4}$$

where $G_{i\omega_n+\mu}$ is the full real-space Green's function, while $\Xi_{i\omega_n+\mu}$ is the same for a system without the polymer-induced potential perturbation $\mathbf{U}$ and $\beta = 1/(k_B T)$. Explicitly,

$$G_{i\omega_n+\mu} = \Xi_{i\omega_n+\mu} + \Xi_{i\omega_n+\mu}\mathbf{U}\left(1 - \Xi_{i\omega_n+\mu}\mathbf{U}\right)^{-1} \Xi_{i\omega_n+\mu} \,. \tag{5}$$

By taking the diagonal elements of $G_{i\omega_n+\mu}$ and replacing $i\omega_n \to \omega + i0^+$ allows us to write down the spectral function:

$$\mathcal{A}_\omega\left(\mathbf{r}_j\right) = -2\mathrm{Im}\left[\Xi_{\omega+\mu} + \Xi_{\omega+\mu}\mathbf{U}\left(1 - \Xi_{\omega+\mu}\mathbf{U}\right)^{-1} \Xi_{\omega+\mu}\right]_{jj} \,, \tag{6}$$

where the energy ω is measured from the Fermi level.

Note that at this point, Ξ and $\mathbf{U}$ are $N \times N$ matrices. Naturally, as $N \to \infty$, the problem becomes intractable numerically. However, we now show that the expression simplifies considerably when one takes into account the structure of $\mathbf{U}$. We write

$$\left[\Xi_{\omega+\mu} + \Xi_{\omega+\mu}\mathbf{U}\left(1 - \Xi_{\omega+\mu}\mathbf{U}\right)^{-1} \Xi_{\omega+\mu}\right]_{jj} = \left[\Xi_{\omega+\mu}\right]_{jj} + \sum_{klm} \left[\Xi_{\omega+\mu}\right]_{jk} \mathbf{U}_{kl} \left[1 - \Xi_{\omega+\mu}\mathbf{U}\right]^{-1}_{lm} \left[\Xi_{\omega+\mu}\right]_{mj} \,. \tag{7}$$

One can see here that k and l in the summation only include states that have non-vanishing elements in $\mathbf{U}$. In addition, expanding $\left[1 - \Xi_{i\omega_n+\mu}\mathbf{U}\right]^{-1}_{lm}$ as a geometric series, makes it clear that m also must correspond to a state with a finite elements in $\mathbf{U}$. This allows us to write

$$\left[\Xi_{\omega+\mu} + \Xi_{\omega+\mu}\mathbf{U}\left(1 - \Xi_{\omega+\mu}\mathbf{U}\right)^{-1} \Xi_{\omega+\mu}\right]_{jj} = \Xi^{jj}_{\omega+\mu} + \begin{pmatrix}\Xi^{j1}_{\omega+\mu} & \Xi^{j2}_{\omega+\mu} & \ldots\end{pmatrix} \tilde{\mathbf{U}} \left(1 - \tilde{\Xi}_{\omega+\mu}\tilde{\mathbf{U}}\right)^{-1} \begin{pmatrix}\Xi^{1j}_{\omega+\mu} \\ \Xi^{2j}_{\omega+\mu} \\ \vdots\end{pmatrix} \,, \tag{8}$$

where the tilde indicates that only the unit cells with the potential perturbation are included and

$$\Xi^{jk}_z = \sum_{lm} \left[\Xi_z\right]_{jk} = \sum_{lm} \Theta_{jl} \left(z - H_{\mathbf{Q}}\right)^{-1}_{lm} \Theta^\dagger_{mk} = \sum_{l} \Theta_{jl} \left(z - H_{\mathbf{Q}}\right)^{-1}_{ll} \Theta^\dagger_{lk} = \frac{1}{N} \sum_{\mathbf{q}} \frac{e^{i\mathbf{q}\cdot(\mathbf{r}_j - \mathbf{r}_k)}}{z - \varepsilon_{\mathbf{q}}} \,. \tag{9}$$

The momentum sum can be turned into an integral

$$\frac{1}{N}\sum_{\mathbf{q}}\frac{e^{i\mathbf{q}\cdot\mathbf{R}}}{z-\hbar^2q^2/(2m_em^*)}=\frac{A}{(2\pi)^2}\oint d\theta\int dq\,q\frac{e^{iqR\cos\theta}}{z-\mathrm{Ry}\times a_0^2q^2/m^*}$$
$$=\frac{1}{\mathrm{Ry}}\frac{A}{2\pi}\int dq\,q\frac{J_0(qR)}{z/\mathrm{Ry}-a_0^2q^2/m^*}=\frac{\sqrt{m^*}}{\mathrm{Ry}}\frac{A/a_0^2}{2\pi}\int du\,u\frac{J_0(\sqrt{m^*}uR/a_0)}{z/\mathrm{Ry}-u^2}\,, \tag{10}$$

where K_0 is the modified Bessel function, A is the area of the unit cell, Ry is the Rydberg energy, and a_0 is the Bohr radius.

If $R\neq 0$, we set the limits of u integration as $[0,\infty)$ to yield

$$\Xi_z^{R\neq 0}=\frac{\sqrt{m^*}}{\mathrm{Ry}}\frac{\tilde{A}}{2\pi}\int_0^\infty du\,u\frac{J_0(\sqrt{m^*}u\tilde{R})}{z/\mathrm{Ry}-u^2}=-\frac{\sqrt{m^*}}{\mathrm{Ry}}\frac{\tilde{A}}{2\pi}K_0\left(\sqrt{m^*}\tilde{R}\sqrt{-\frac{z}{\mathrm{Ry}}}\right)\,, \tag{11}$$

where $\tilde{A}=A/a_0^2$ and $\tilde{R}=R/a_0$.

For $R=0$, the integral diverges and we introduce a cutoff so that $u\in[0,\sqrt{C}]$ so that

$$\Xi_z^{R=0}=\frac{\sqrt{m^*}}{\mathrm{Ry}}\frac{\tilde{A}}{2\pi}\int_0^{\sqrt{C}} du\frac{u}{z/\mathrm{Ry}-u^2}=-\frac{1}{2}\frac{\sqrt{m^*}}{\mathrm{Ry}}\frac{\tilde{A}}{2\pi}\ln\left(1-\frac{C}{z/\mathrm{Ry}}\right)\,. \tag{12}$$

As the final step, we determine the value of C. Using the fact that $-2\mathrm{Im}[\Xi_{\omega+i0}^{R=0}]$ is the spectral function for the pristine system whose integral on $\omega\in(-\infty,\infty)$ yields 2π, we get

$$\int_{-\infty}^{\infty}d\omega\mathrm{Im}\left[\frac{\sqrt{m^*}}{\mathrm{Ry}}\frac{\tilde{A}}{2\pi}\ln\left(1-\frac{C\times\mathrm{Ry}}{\omega+i0}\right)\right]=\frac{\sqrt{m^*}}{\mathrm{Ry}}\frac{\tilde{A}}{2\pi}\times C\times\mathrm{Ry}\times\pi \tag{13}$$

leading to $C=\frac{4\pi}{\tilde{A}\sqrt{m^*}}$. This yields

$$\Xi_z^{R\neq 0}=-\frac{2}{C\mathrm{Ry}}K_0\left(\sqrt{m^*}\tilde{R}\sqrt{-\frac{z}{\mathrm{Ry}}}\right)\,,$$
$$\Xi_z^{R=0}=-\frac{1}{C\mathrm{Ry}}\ln\left(1-\frac{C\mathrm{Ry}}{z}\right)\,. \tag{14}$$

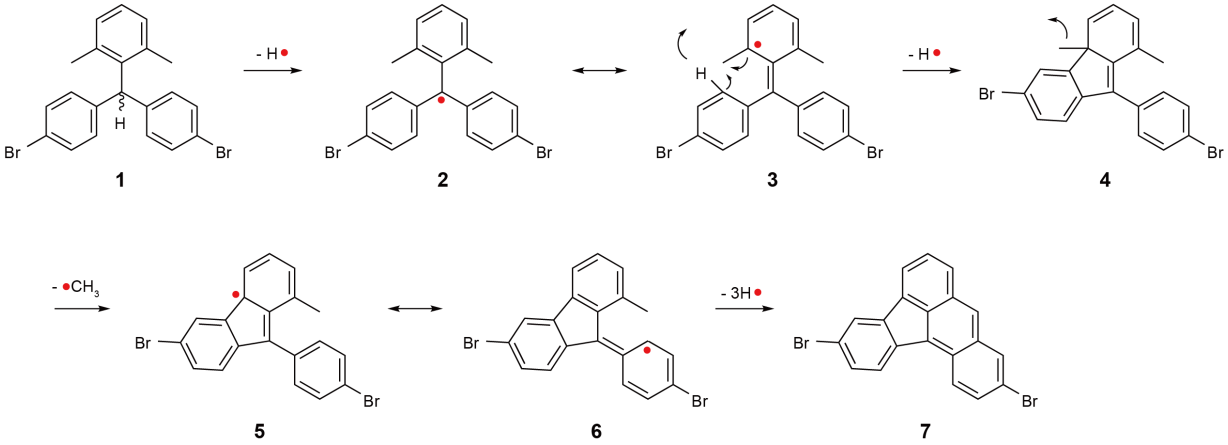

Supplementary Figure 1. Proposed reaction pathway for the formation of pentagonal ring via the demethylation

Supplementary Note 2. Proposed mechanism of the formation of pentagonal ring.

The precursor **1** is expected to undergo the thermally-triggered dehydrogenation, leading to the formation of one radical at the central methylene (**2**). It can be delocalized to dimethylphenyl site for the subsequent bonding with bromophenyl ring to form pentagonal ring (from **3** to **6**) via demethylation. A further dehydrogenation on the other methyl group and the other bromophenyl generates planar **7**. The high chemical activity of monoradical **2** is expected to be the driving force towards the formation of closed-shell compound **7** with planar structure[1].

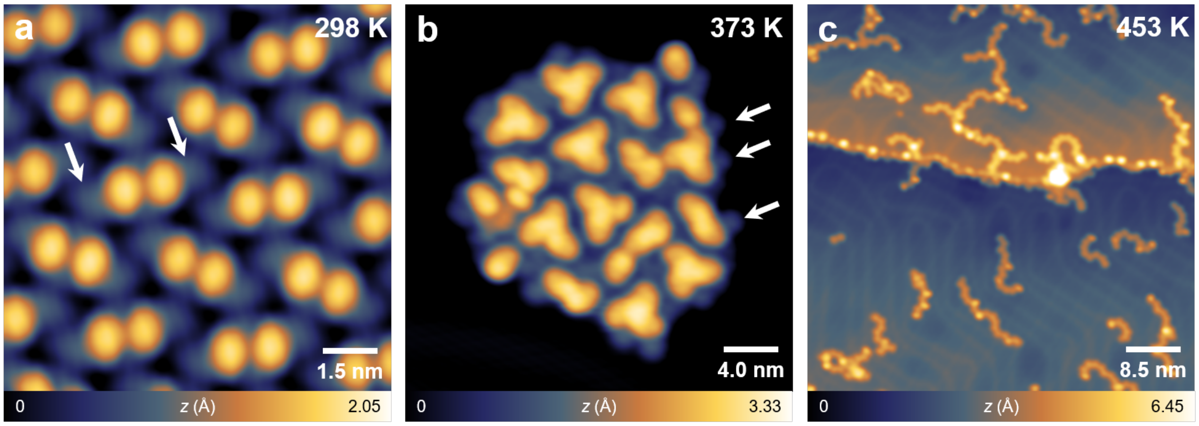

Supplementary Figure 2. **STM images of self-assembled precursor 1, debrominated precursor 1 and polymer chains after Ullmann coupling**. **a**, STM image of self-assembled precursor **1** on Au(111) without annealing. Br substituents appear as small dark dots as highlighted with white arrows ($V = 0.1$ V, $I = 300$ pA). **b**, STM image obtained after annealing precursor **1** on Au(111) at 373 K reveals clusters of debrominated precursors **1** (their nonplanar topology indicates the existence of hydrogens or methyl groups) coexisting with adsorbed Br atoms (highlighted with white arrows) near the edges of clusters ($V = -2.0$ V, $I = 300$ pA). **c**, STM image obtained after annealing precursor **1** on Au(111) at 453 K reveals curved segments and polymer chains derived from incomplete cyclization as indicated by the small bright protrusions ($V = 1.0$ V, $I = 30$ pA).

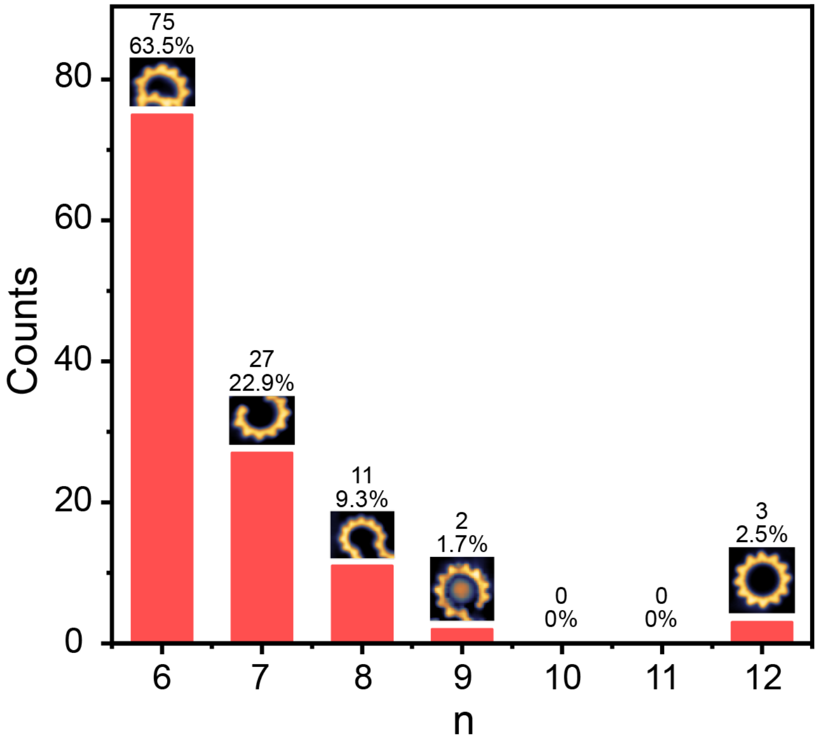

Supplementary Figure 3. Statistic analysis of regular curved ring segments with different numbers (n) of building units (regular curved ring segments and complete rings with n > 12 were not observed).

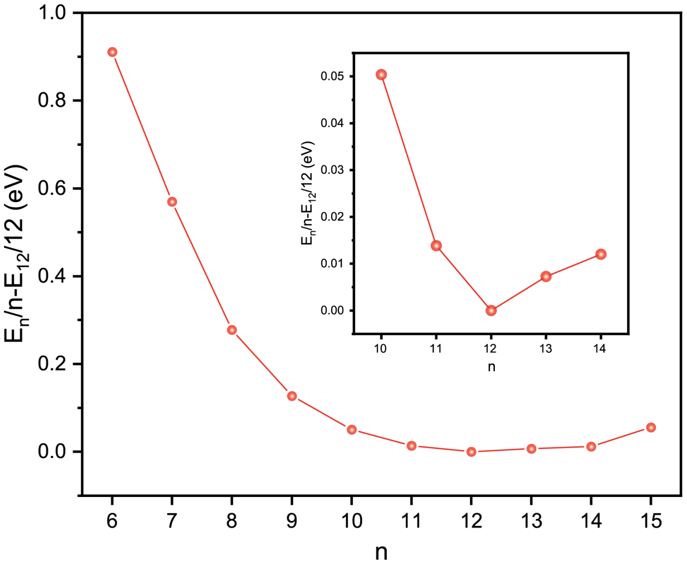

Supplementary Figure 4. Plot of energy differences between total energy divided by number of units and the reference energy of 12-unit macrocycle. Inset shows the energy differences of neighboring points (n = 10, 11, 13, 14) relative to the global minimum (n = 12).

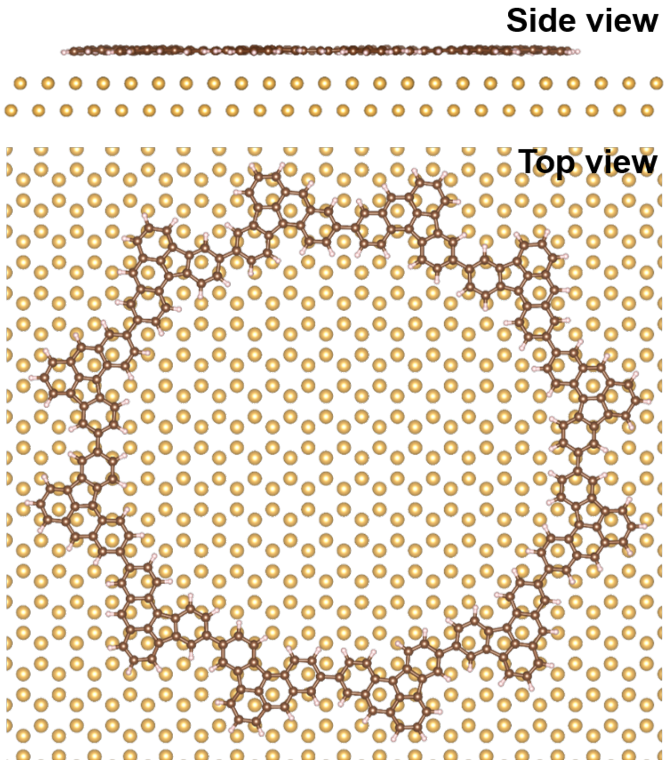

Supplementary Figure 5. Side view (top) and top view (bottom) of the DFT-relaxed structure of 12-OQC on Au(111).

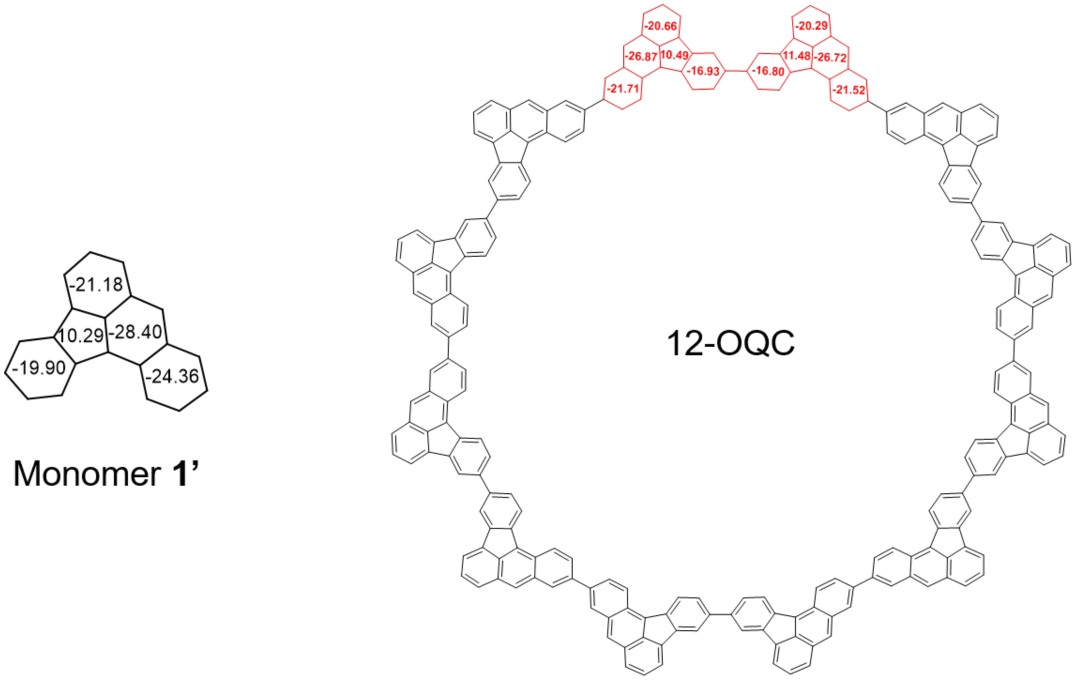

Supplementary Figure 6. NICS calculations of a hydrogenated version of monomer **1'** (left) and 12-unit macrocycle (right). Monomer and macrocycle show similar NICS values and localized aromaticity. NICS values were calculated using the standard GIAO procedure (NMR pop=NCSall)[2] at the RB3LYP[3]/6-31G(d,p)[4] level of theory and the NICS$(1)_{zz}$ value for the reference benzene is -29.69 ppm[5].

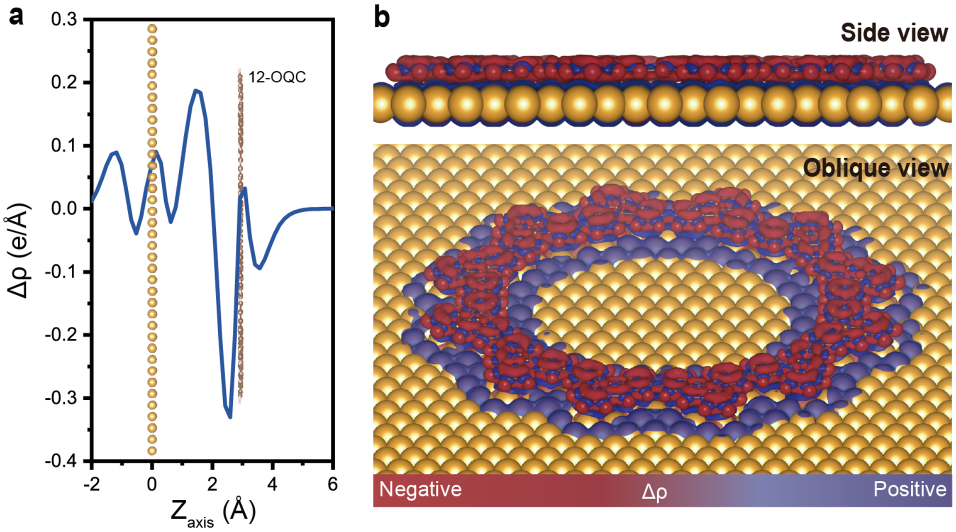

Supplementary Figure 7. **DFT-calculated charge redistribution of a 12-OQC on Au(111). a**, Projection of the charge redistribution of 12-OQC on Au(111) along the z-axis ($\Delta\rho = \rho_{\text{total}} - \rho_{\text{surface}} - \rho_{\text{molecule}}$). **b**, 3D isosurface of the electronic density differences with an isovalue of ±0.001 e/Å^3 of 12-OQC on Au(111) surface (upper panel and bottom panel are side and oblique view, respectively). Red and purple regions in 3D plots represent charge depletion and accumulation, respectively.

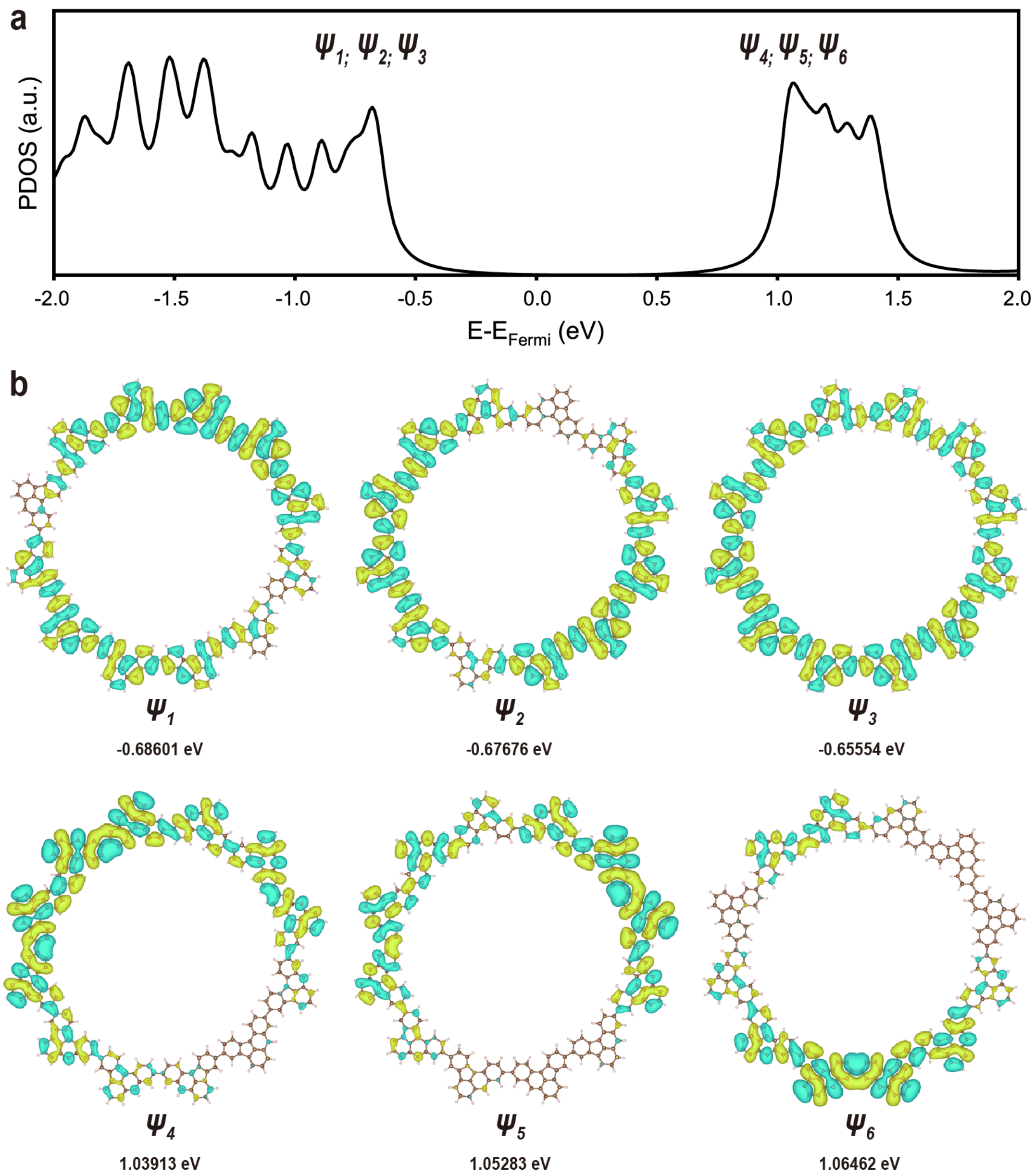

Supplementary Figure 8. **Orbital structure of a freestanding 12-OQC.** **a**, The projected density of states (PDOS) of 12-OQC calculated by BLYP functional. **b**, Spatial distribution of ψ_1 (-0.68601 eV), ψ_2 (-0.67676 eV), ψ_3 (-0.65554 eV), ψ_4 (1.03913 eV), ψ_5 (1.05283 eV), ψ_6 (1.06462 eV) orbitals of 12-OQC. Isosurface value: 0.015 e/Å^3.

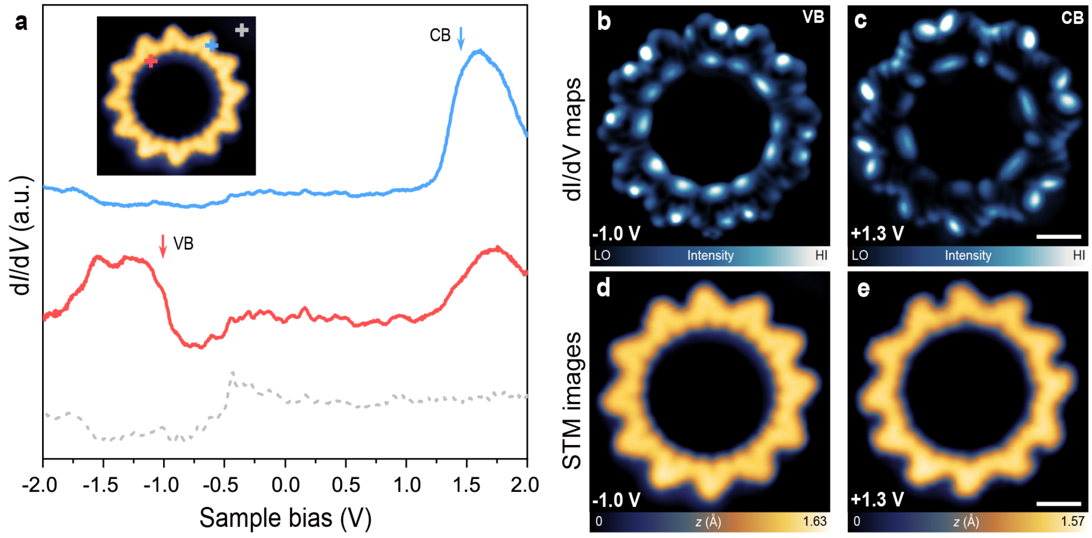

Supplementary Figure 9. **Electronic structure characterization of a second 12-OQC.** **a**, Point dI/dV spectra acquired over different sites of 12-OQC and Au(111) substrate. dI/dV curves taken at the pentagonal ring (blue curve), inner edge of 12-OQC (red curve), and taken on Au(111) (grey dashed curve). **b**,**c**, Constant-current dI/dV maps recorded at the energy positions of the VB (−1.0 V) and CB (+1.3 V) of 12-OQC, respectively. **d**,**e**, The corresponding STM images of **b**,**c** collected simultaneously with constant current dI/dV maps (I = 2 nA for **b**-**e**). Scale bar: 1 nm.

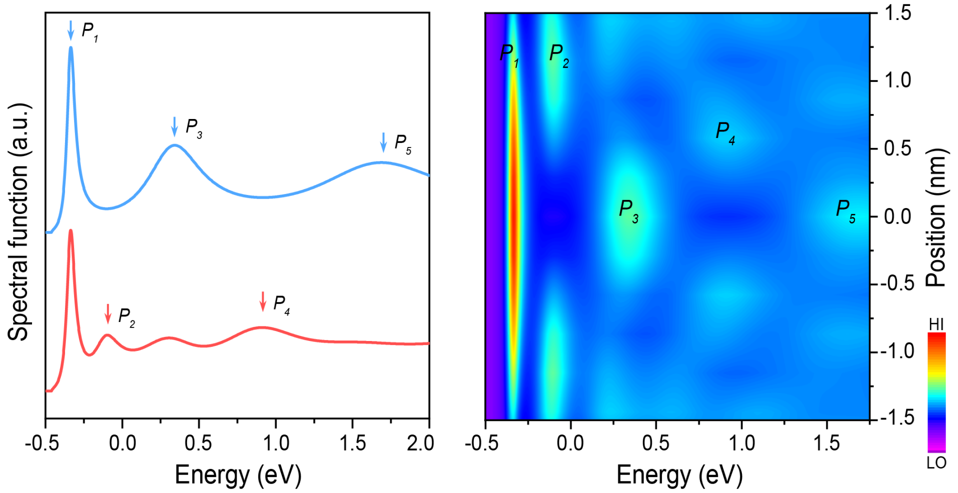

Supplementary Figure 10. Calculated position-dependent spectral function over different sites inside 12-OQC (left). Blue and red curve taken at the centre and 0.6 nm away from the centre of simulated model. Color-coded simulated spectral function curves (spaced by 0.15 nm) taken across the pore of potential corral in gold unit cells (right).

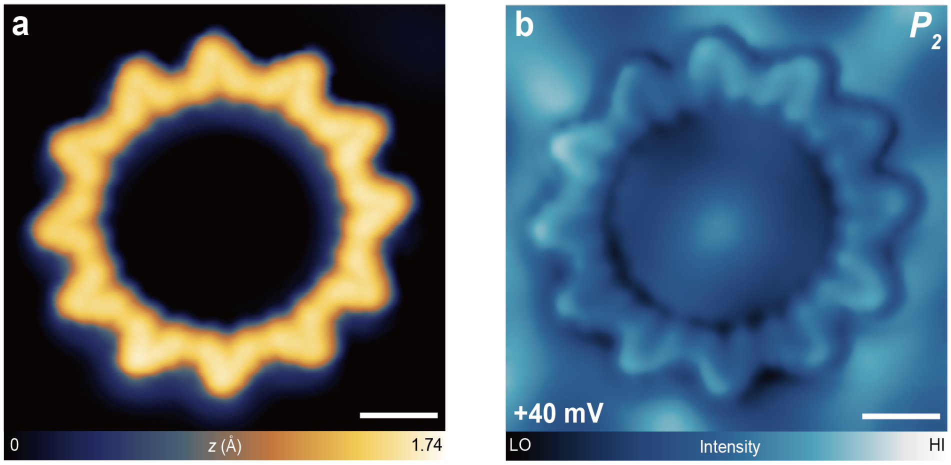

Supplementary Figure 11. **Resonance state (P_2) inside the 12-OQC. a**, The corresponding STM image of **b** collected simultaneously with constant-current dI/dV map (V = +40 mV, I = 1 nA) of 12-OQC. **b**, Constant-current dI/dV map recorded at +40 mV. Scale bar: 1 nm.

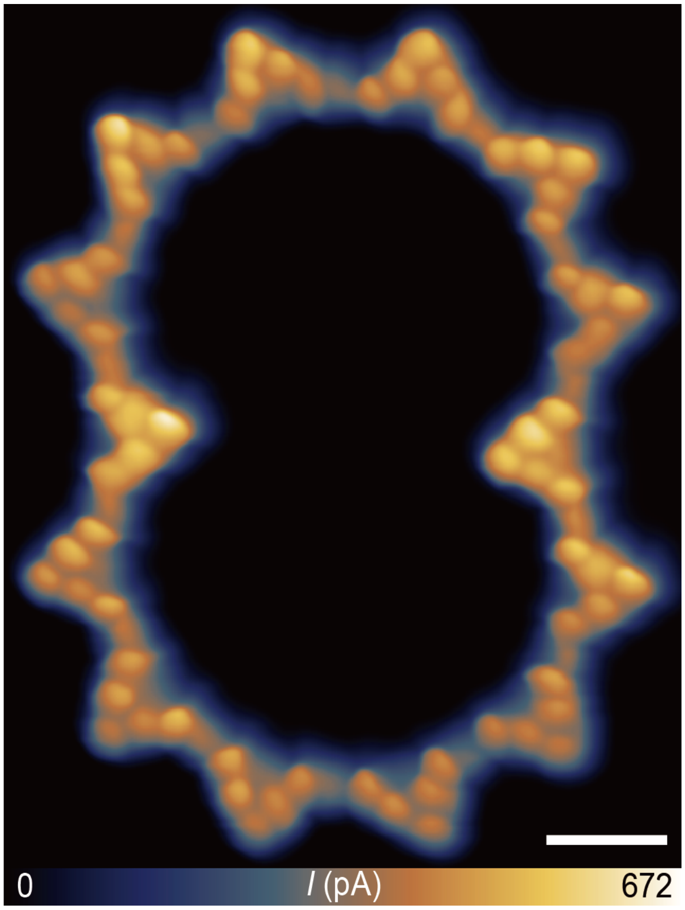

Supplementary Figure 12. BR-STM image of a symmetric COS-OQC (V = 10 mV, Δz = −1.5 Å; set point prior to turn off feedback, V = 20 mV, I = 600 pA). Scale bar: 1 nm.

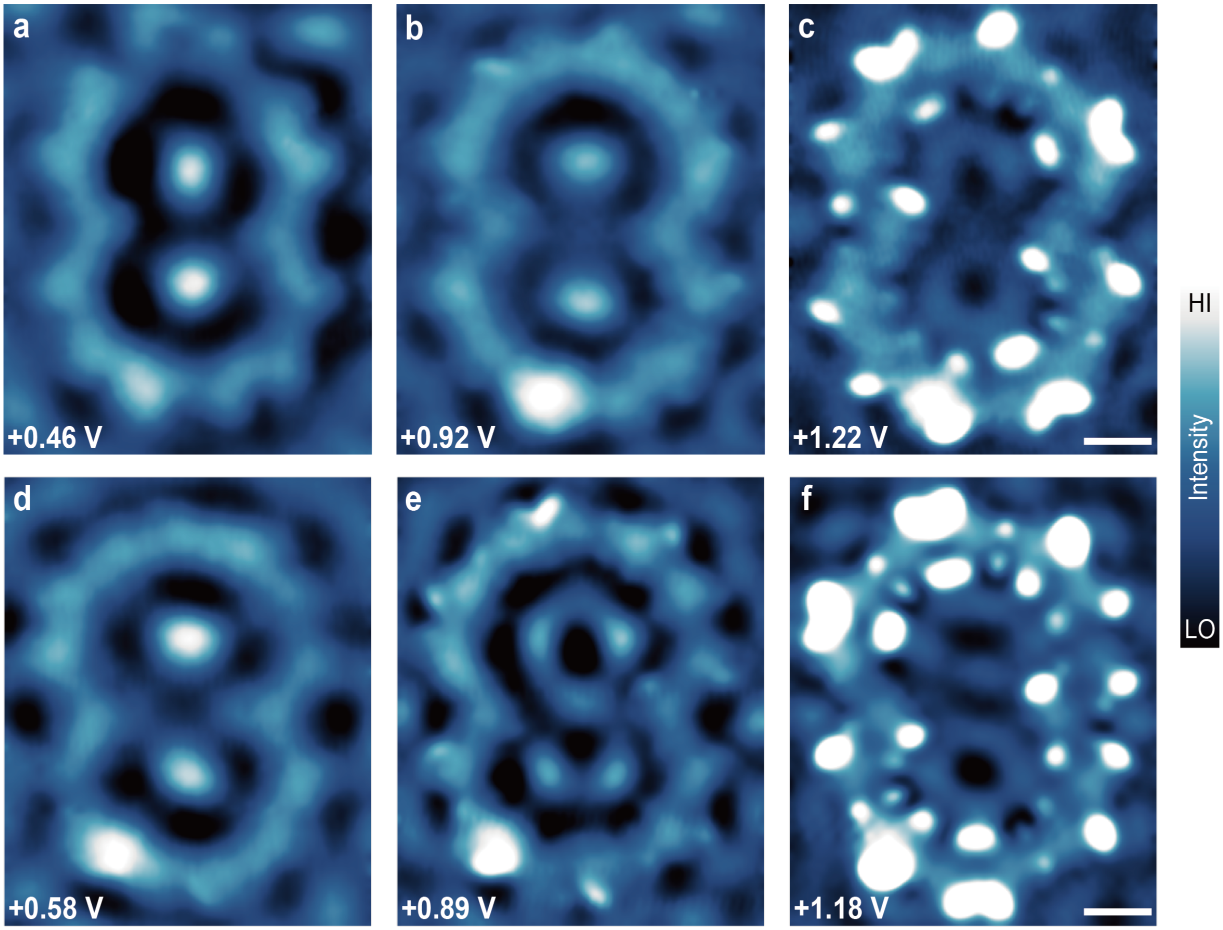

Supplementary Figure 13. **Characterization of quantum resonance states in symmetric and asymmetric COS-OQC. a-c**, Constant-current dI/dV maps of a symmetric COS-OQC recorded at different energy positions (I = 1 nA for **a**; I = 1.5 nA for **b**,**c**). **d-f**, Constant-current dI/dV maps of an asymmetric COS-OQC recorded at different energy positions (I = 1 nA for **d**; I = 1.5 nA for **e**,**f**). Scale bar: 1 nm.

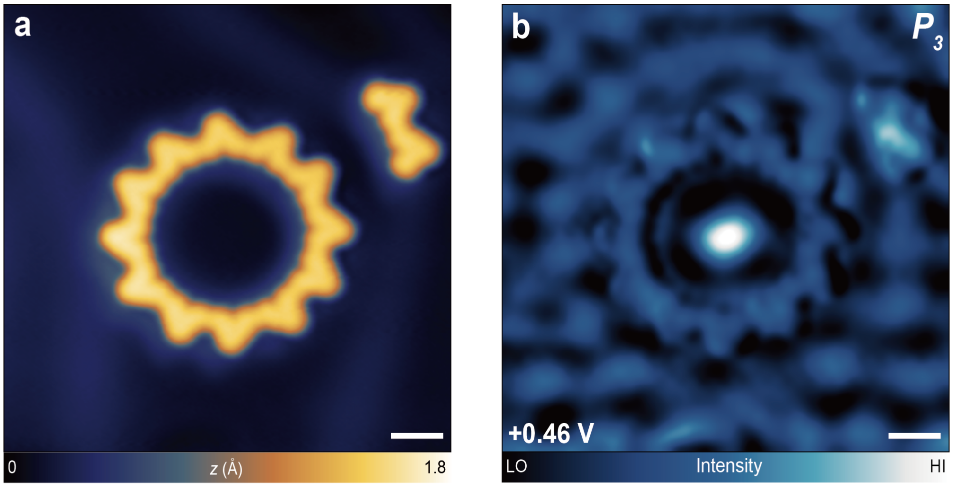

Supplementary Figure 14. **Scattered wave patterns surrounding the 12-OQC. a**, The corresponding STM image of **b** collected simultaneously with constant-current dI/dV map (V = +0.46 V, I = 1.2 nA) of 12-OQC. **b**, Constant-current dI/dV map recorded at +0.46 V (P_3). Scale bar: 1 nm.

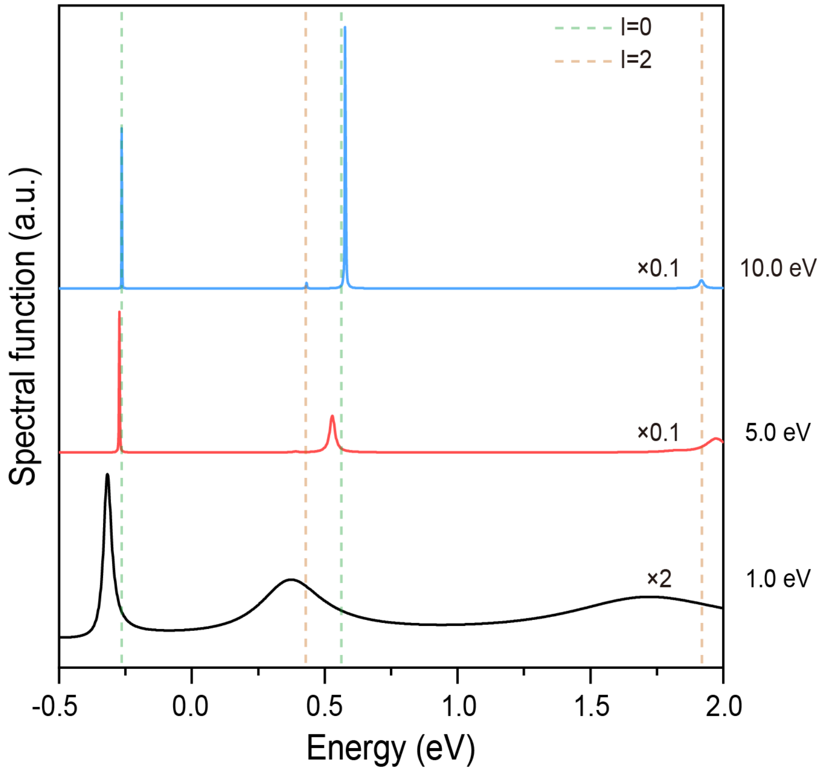

Supplementary Figure 15. **Comparison between resonance states in quantum corrals with different barrier heights and bound states.** Calculated spectral function curves taken at the centre of simulated 12-OQC model with barrier heights of 1.0 eV (black curve), 5.0 eV (red curve) and 10.0 eV (blue curve), respectively. The dashed lines present the energy positions of bound states in the quantum well with the same geometry when the angular momentum $l = 0$ (green dashed lines) and 2 (orange dashed lines).

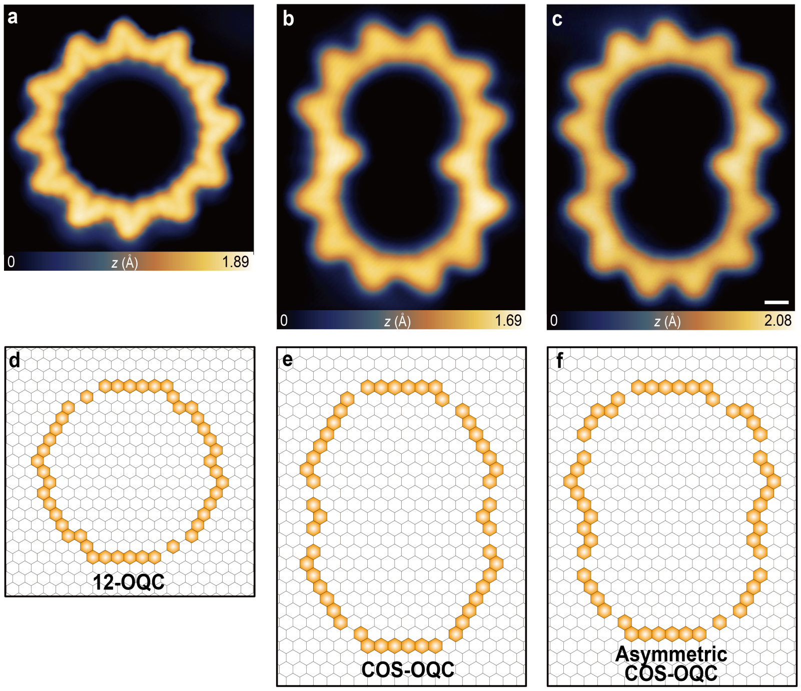

Supplementary Figure 16. **The positions of repulsive potential scatters placed over Au unit cells.** **a**, STM image of 12-OQC ($V = -0.26$ V, $I = 1$ nA). **b**, STM image of a symmetric COS-OQC ($V = -0.32$ V, $I = 1.5$ nA). **c**, STM image of an asymmetric COS-OQC ($V = -0.3$ V, $I = 1.5$ nA). **d-f**, Corresponding simulation models of repulsive potential scatter placed over Au unit cells for 12-OQC, COS-OQC and asymmetric COS-OQC, respectively (the unit cells in orange color indicate the positions where we included the repulsive potential scatters). Scale bar: 5 Å.

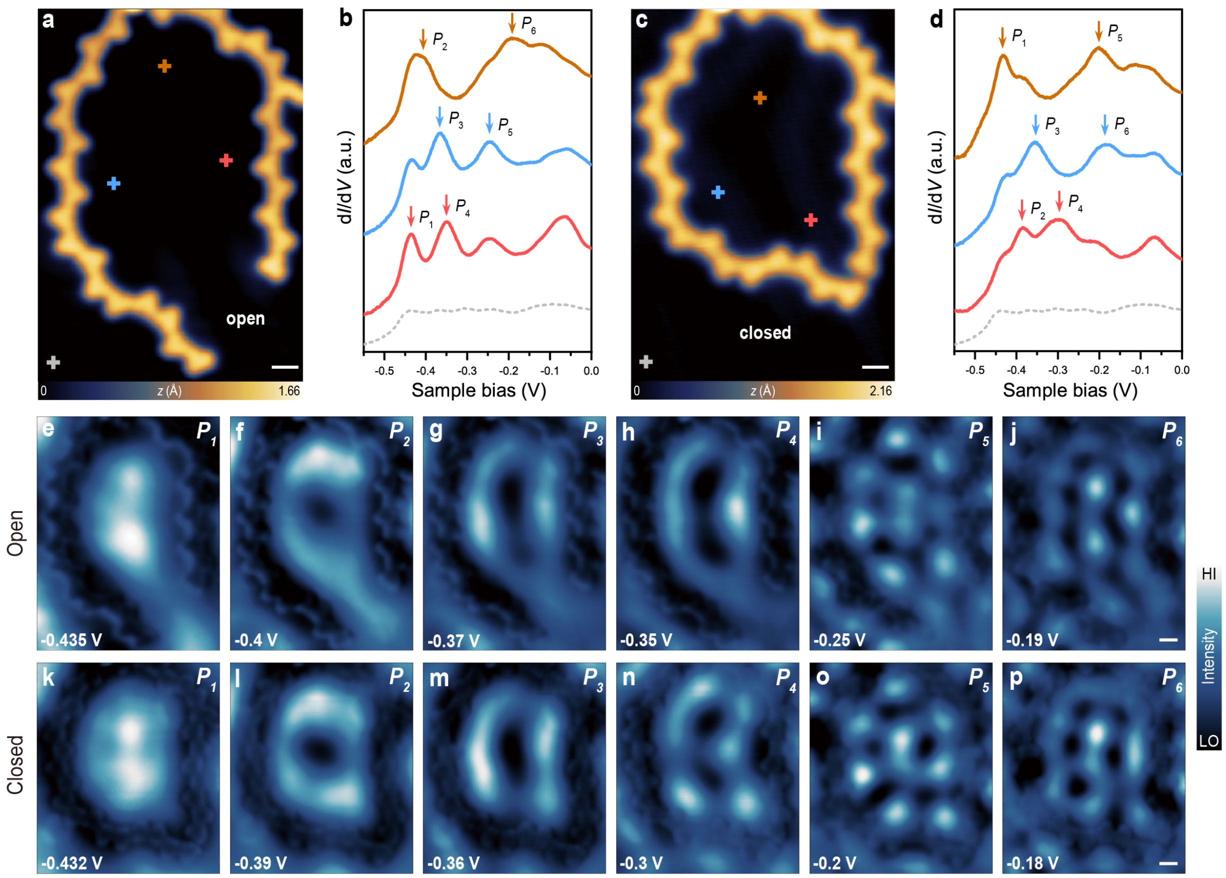

Supplementary Figure 17. **Control the geometries of OQC by tip manipulation to tune their resonance states.** **a**, STM image of open OQC before manipulation ($V = -0.37$ V, $I = 1$ nA). **b**, Point dI/dV spectra acquired over different sites of OQC and Au(111) substrate. dI/dV curves taken at the position of red cross in **a** (red curve), blue cross in **a** (blue curve), orange cross in **a** (orange curve), and taken on Au(111) (grey dashed curve). **c**, STM image of closed OQC after manipulation ($V = -0.432$ V, $I = 1$ nA). **d**, Point dI/dV spectra acquired over different sites of closed OQC and Au(111) substrate. dI/dV curves taken at the position of red cross in **c** (red curve), blue cross in **c** (blue curve), orange cross in **c** (orange curve), and taken on Au(111) (grey dashed curve). **e-j**, Constant-current dI/dV maps recorded at the energy positions of the P_1 (-0.435 V), P_2 (-0.4 V), P_3 (-0.37 V), P_4 (-0.35 V), P_5 (-0.25 V) and P_6 (-0.19 V) of OQC, respectively ($I = 1$ nA for **e-h**; $I = 800$ pA for **i**,**j**). **k-p**, Constant-current dI/dV maps recorded at the energy positions of the P_1 (-0.432 V), P_2 (-0.39 V), P_3 (-0.36 V), P_4 (-0.3 V), P_5 (-0.2 V) and P_6 (-0.18 V) of closed OQC, respectively ($I = 1$ nA for **k-n**; $I = 800$ pA for **o**,**p**). Scale bar: 1 nm.

Supplementary Note 3. Tuning the quantum resonance states by tip manipulation.

To conduct tip manipulation[6,7], we first positioned the STM tip over the terminus of OQC with a set point of $V = 1$ mV, $I = 100$ pA and then switched off the feedback loop. The tip-sample distance was subsequently reduced by 1.5 Å, at which an abrupt increase in tunnelling current occurs, suggesting the contact of tip apex with the terminus of OQC. After retracting the tip by 200 pm, a controllable STM lateral manipulation was conducted by moving the tip along a defined trajectory (Supplementary Figure 17c). We then performed dI/dV measurements to probe the resonance states before and after tip manipulation. As shown in Supplementary Figure 17b,d, a series of resonance states were revealed in the corresponding dI/dV spectra acquired at the positions indicated by the crosses with different colors in Supplementary Figure 17a,c, which are labelled as P_1-P_6 from low energy to high energy. We then carried out dI/dV mapping to probe the spatial distribution of these resonance states. By comparing the patterns of dI/dV maps of original OQC in Supplementary Figure 17e-j with their corresponding dI/dV maps after manipulation in Supplementary Figure 17k-p, the similar hot spots (P_1, P_5 and P_6) and ring-like features (P_2, P_3 and P_4) observed in these two OQCs suggest they are originated from the same resonance state. As expected, the energetic positions of these states all shift to higher energy after tip manipulation. This is because a smaller size of corral after tip manipulation is expected to produce the quantum resonance states at high energy position. This proves that quantum states in OQCs could be further engineered *via* tip manipulation.

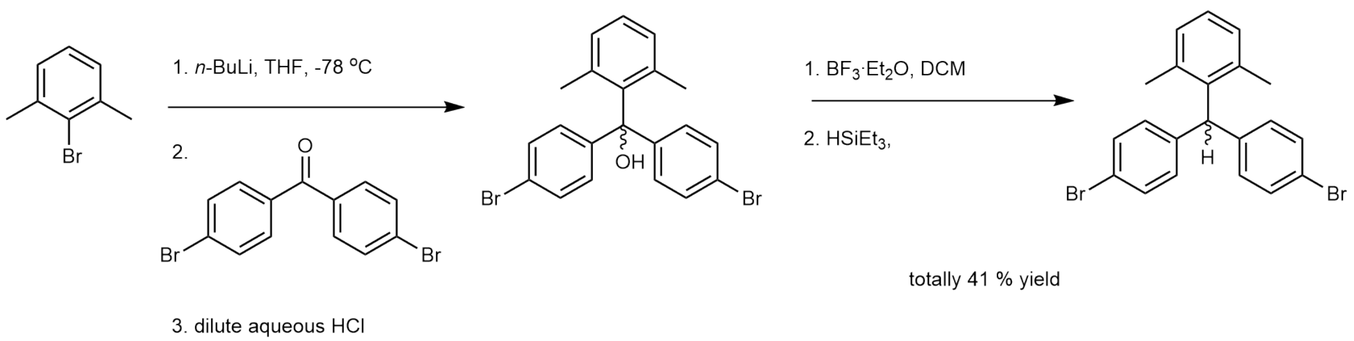

Supplementary Figure 18. Synthetic route of precursor **1**

Supplementary Note 4. Synthetic route.

n-BuLi (1.0 ml, 2.0 mmol, 2M) was added dropwise into the solution of 2-bromo-1,3-dimethylbenzene (0.30 ml, 2.2 mmol) in dry THF (20 ml) at -78 °C, and the solution was stirred at this temperature for 2 hrs. Then, bis(4-bromophenyl)methanone (750 mg, 2.2 mmol) was added into the solution. After stirring for 2 hrs at -78 °C, the mixture was gradually warmed up to room temperature. Another 2 hrs later, dilute aqueous HCl solution (5 ml, 1M) was added to quench the reaction. Then, diethyl ether (30 ml) was added and the organic layer was washed with brine for three times and dried over anhydrous sodium sulfate. The crude product was directly used for next step after evaporating off the solvent. The crude product was dissolved in dry dichloromethane (DCM, 30 ml), and $BF_3{\cdot}Et_2O$ (0.30 ml, 2.3 mmol) was added into the solution. 30 mins later, triethylsilane (0.35 ml, 2.2 mmol) was dropwise into the mixture. 1 hr later, the reaction was quenched with triethylamine (0.5 ml) and the solution was washed with brine for three times and dried over anhydrous sodium sulfate. After removing the solvent, the crude product was purified by column chromatography with DCM/hexane as eluent (1/8, v/v) to afford precursor **1** (350 mg) in 41% yield.

^{1}H NMR (500 MHz, $CDCl_3$): δ 7.40 (d, J = 8.5 Hz, 4H), 7.12 (t, J = 7.5 Hz, 1H), 7.03 (d, J = 7.5 Hz, 2H), 6.94 (d, J = 8.6 Hz, 4H), 5.89 (s, 1H), 2.01 (s, 6H);

^{13}C NMR (125 MHz, $CDCl_3$): δ 140.73, 138.81, 137.46, 131.37, 130.91, 129.55, 127.11, 120.07, 50.25, 22.06.

HRMS analysis (m/z): $[M]^+$ calcd. for $C_{19}H_{15}NBr$, 427.977; found, 427.9775 (error: 1.17 ppm).

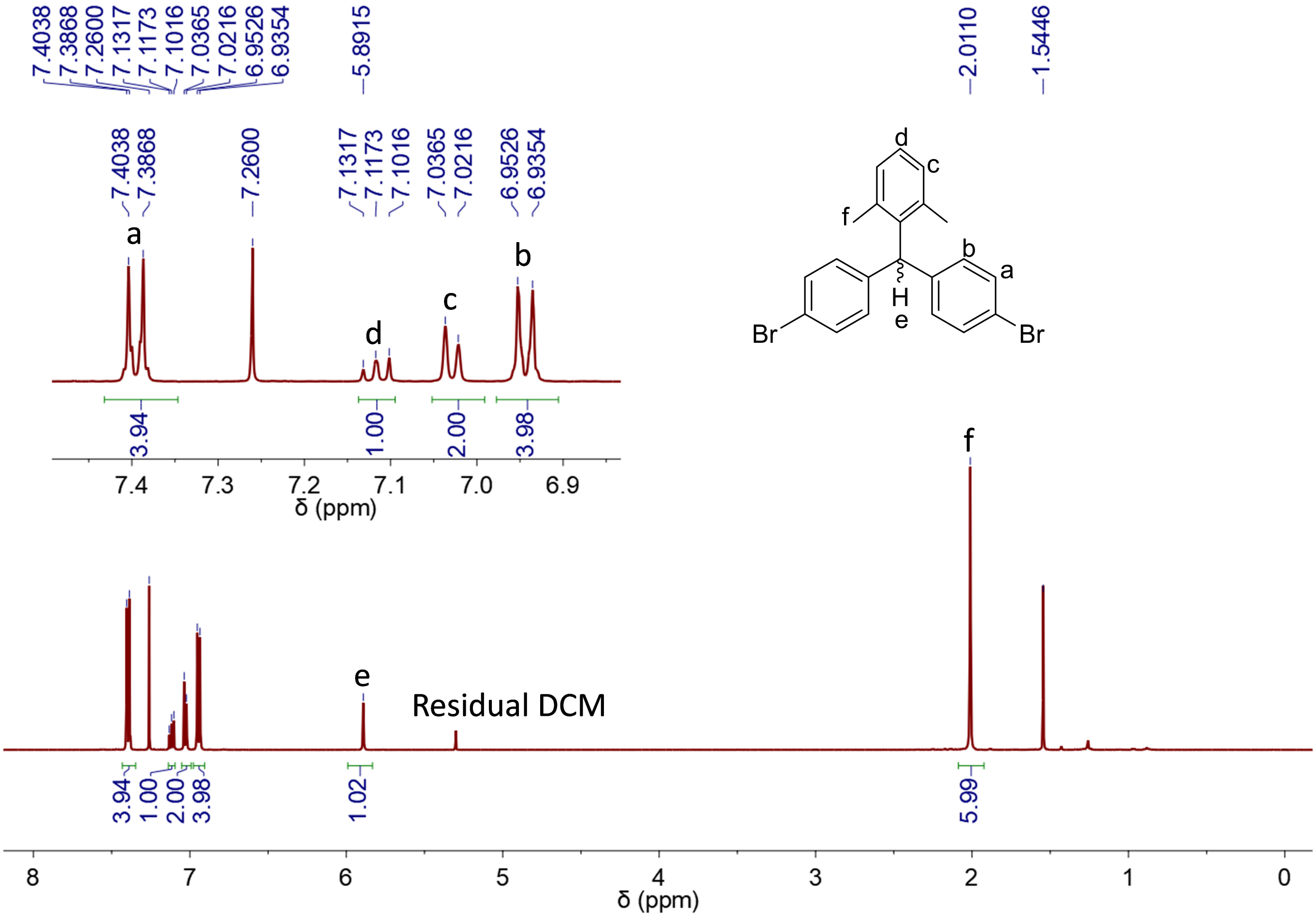

Supplementary Figure 19. ^{1}H NMR spectrum of precursor **1** (500 MHz, $CDCl_3$).

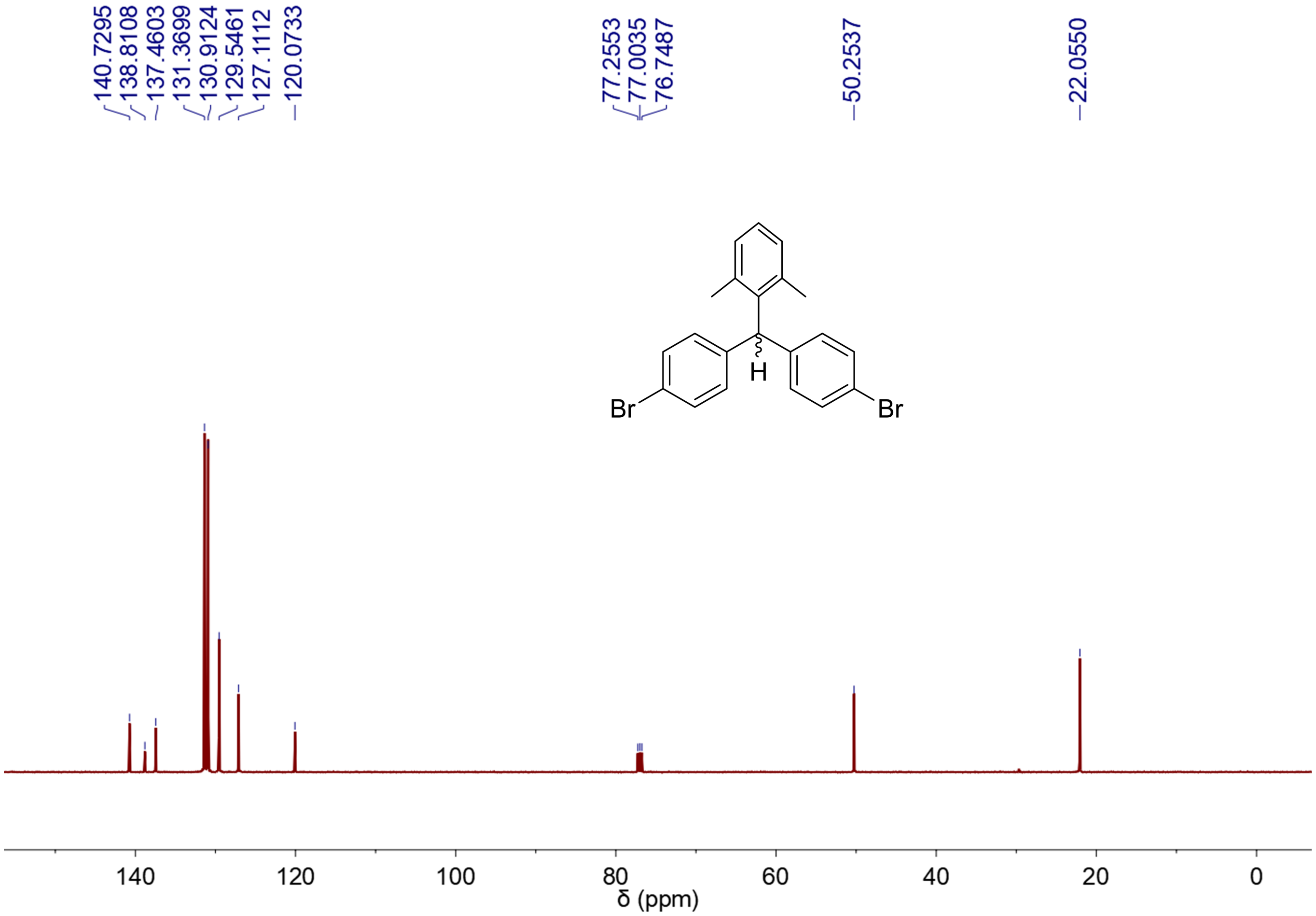

Supplementary Figure 20. ^{13}C NMR spectrum of precursor **1** (125 MHz, $CDCl_3$).

Mass Spectrum SmartFormula Report

**Sample Name**	1	**Data File**	D:\Chem\2021\202104\20210416\1.D
**Instrument Name**	Agilent 7200 GC-QTOF	**IRM Calibration Status**	Success
**Acq Method**	EIHR_CalValve.ei.m	**Acquired Time**	16/4/2021 3:13:11 PM (UTC+08:00)
**Comment**	A/P Chi Chunyan	**Operator**	

Meas. m/z	#	Formula	Calc. Mass	Err [ppm]
427.9775	1	C21 H18 [79Br]2	427.977	1.17

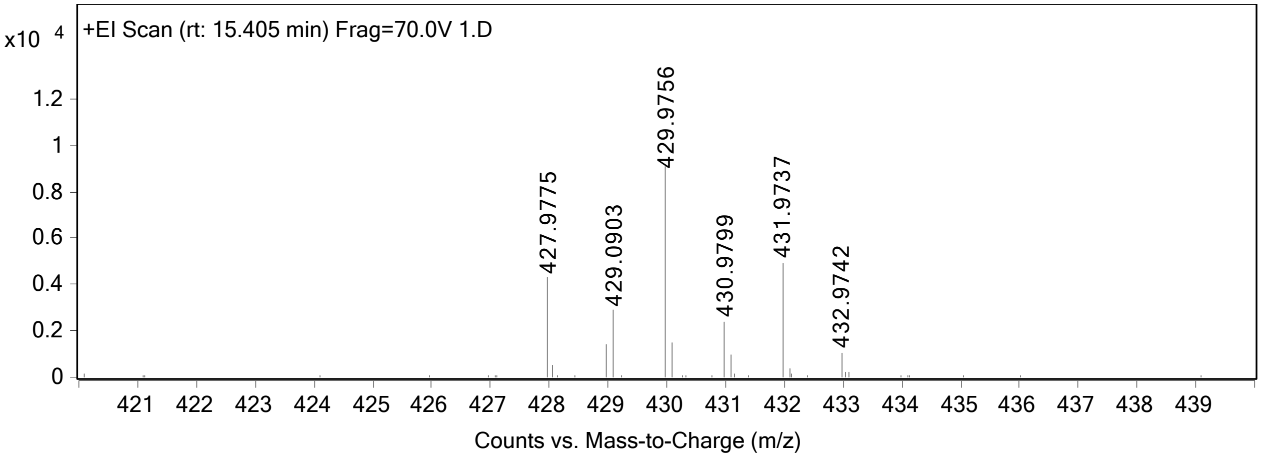

Page 1 of 1 Printed at 4:50 PM on 16-Apr-2021

Supplementary Figure 21. HR mass spectrum (EI) of precursor **1**.

Supplementary References

[1] Song, S. *et al.* Real-Space Imaging of a Single-Molecule Monoradical Reaction. *J. Am. Chem. Soc.* **142**, 13550–13557 (2020).

[2] Chen, Z., Wannere, C. S., Corminboeuf, C., Puchta, R. & Schleyer, P. V. R. Nucleus-independent chemical shifts (nics) as an aromaticity criterion. *Chem. Rev.* **105**, 3842–3888 (2005).

[3] Lee, C., Yang, W. & Parr, R. G. Development of the colle-salvetti correlation-energy formula into a functional of the electron density. *Phys. Rev. B* **37**, 785 (1988).

[4] Hehre, W. J., Ditchfield, R. & Pople, J. A. Self—consistent molecular orbital methods. xii. further extensions of gaussian—type basis sets for use in molecular orbital studies of organic molecules. *J. Chem. Phys.* **56**, 2257–2261 (1972).

[5] Schleyer, P. v. R., Maerker, C., Dransfeld, A., Jiao, H. & van Eikema Hommes, N. J. Nucleus-independent chemical shifts: a simple and efficient aromaticity probe. *J. Am. Chem. Soc.* **118**, 6317–6318 (1996).

[6] Michnowicz, T. *et al.* Controlling Single Molecule Conductance by a Locally Induced Chemical Reaction on Individual Thiophene Units. *Angew. Chem. Int. Ed.* **59**, 6207–6212 (2020).

[7] Joachim, C., Gimzewski, J. K., Schlittler, R. R. & Chavy, C. Electronic transparence of a single C60 molecule. *Phys. Rev. Lett.* **74**, 2102–2105 (1995).
